# Microbiome Variation Across Two Hemlock Species With Hemlock Woolly Adelgid Infestation

**DOI:** 10.3389/fmicb.2020.01528

**Published:** 2020-07-07

**Authors:** Nicholas C. Dove, Timothy J. Rogers, Christy Leppanen, Daniel Simberloff, James A. Fordyce, Veronica A. Brown, Anthony V. LeBude, Thomas G. Ranney, Melissa A. Cregger

**Affiliations:** ^1^Biosciences Division, Oak Ridge National Laboratory, Oak Ridge, TN, United States; ^2^Department of Microbiology, The University of Tennessee, Knoxville, Knoxville, TN, United States; ^3^Department of Ecology & Evolutionary Biology, The University of Tennessee, Knoxville, Knoxville, TN, United States; ^4^Department of Horticultural Science, North Carolina State University, Mills River, NC, United States

**Keywords:** 16S rRNA, epiphyte, ITS, microbial ecology, plant-microbe interactions, plant pathology, phyllosphere, rhizosphere

## Abstract

The hemlock woolly adelgid (*Adelges tsugae*, HWA), an invasive insect, is devastating native hemlock populations in eastern North America, and management outcomes have so far had limited success. While many plant microbiomes influence and even support plant immune responses to insect herbivory, relatively little is known about the hemlock microbiome and its interactions with pathogens or herbivores such as HWA. Using 16S rRNA and ITS gene amplicon sequencing, we characterized the needle, branch, root, and rhizosphere microbiome of two hemlock species, *Tsuga canadensis* and *T. sieboldii*, that displayed low and high levels of HWA populations. We found that both archaeal/bacterial and fungal needle communities, as well as the archaeal/bacterial branch and root communities, varied in composition in both hemlock species relative to HWA population levels. While host species and plant-associated habitats explained a greater proportion of the variance in the microbiome than did HWA population level, high HWA populations were associated with enrichment of 100 likely fungal pathogen sequence variants across the four plant-associated habitats (e.g., needle, branch, root, rhizosphere) compared to trees with lower HWA populations. This work contributes to a growing body of literature linking plant pathogens and pests with the changes in the associated plant microbiome and host health. Furthermore, this work demonstrates the need to further investigate plant microbiome effects across multiple plant tissues to understand their influences on host health.

## Introduction

A growing body of literature recognizes that microorganisms living inside or in close association with plant tissues are integral to plant health and survival (Compant et al., 2005; Santoyo et al., 2016). In some cases, microorganisms can increase their hosts’ resistance to insect herbivory (Pineda et al., 2017) by affecting plant secondary metabolism (Badri et al., 2013; Hubbard et al., 2019). Plant inoculation with foliar fungal isolates has been shown to reduce herbivory by virtue of fungal metabolites toxic to insects (Tibbets and Faeth, 1999), by fungi acting directly on herbivores as insect pathogens (Marcelino et al., 2008), or by “priming” production of salicylic and jasmonic acids used in plant resistance to pests and pathogens (Thaler et al., 2010). However, the extent and mechanisms of microbiome-induced plant pathogen or herbivore resistance are not broadly understood because these services are primarily studied in model plants and important agricultural species such as *Arabidopsis thaliana* (Badri et al., 2013), *Gossypium* (Karban et al., 1987), and *Allium cepa* (Muvea et al., 2014) and less often in trees such as *Populus* (Busby et al., 2013). Interestingly, research also points toward the influence of herbivorous arthropods (mites) on the leaf endosphere microbiome and in particular the fungal pathogens *Melampsora* (Busby et al., 2016) and *Drepanopeziza* (Busby et al., 2019).

Expanding our understanding of the reciprocal influences of insect and arthropod herbivores and plant host microbiomes could be particularly useful in instances where plants that are especially important to ecosystem health are under threat. For example, eastern hemlock (*Tsuga canadensis*) is a foundational species in eastern North American forests (Ellison et al., 2005), yet comparatively little is known about the hemlock microbiome and its interactions with pathogens or herbivores, such as the hemlock woolly adelgid (HWA, *Adelges tsugae*), which is currently devastating native hemlock populations (Eschtruth et al., 2013). The HWA often feeds on young hemlock branches where needles intersect the branch (McClure, 1987), however, the HWA can also be found on the hemlock trunk with unknown consequences for the tree (Oten, 2011; Leppanen et al., 2019). Feeding at the needle base prevents nutrients required for growth from reaching the needles, causing them to discolor and desiccate (Young et al., 1995; McClure et al., 1996; Havill et al., 2016b). The HWA does not appear to harm hemlock species within its native range of Asia and northwestern North America (Oten et al., 2014). However, in the mid 20th century, the HWA arrived in the eastern United States (USA) with the introduction of ornamental hemlocks, and it has since spread from northern Georgia to southern Nova Scotia (Kantola et al., 2019). Hemlock loss can have ecosystem-level effects owing to their foundational role in some eastern mixed hardwood forests. For instance, they provide habitat for many animals (Yamasaki et al., 2000), moderate diel fluctuations in temperature and moisture that improves stream habitats for many invertebrates (Snyder et al., 2002), and slow biogeochemical cycling, preventing stream eutrophication (Jenkins et al., 1999).

The use of chemical control to manage HWA is effective (Silcox, 2002) but not sustainable, and biological control has not yet proven successful in lowering hemlock mortality (Havill et al., 2016b). Resistance or tolerance to HWA in hemlocks from within the native range of the HWA and in some apparently resistant stands in its invasive range is also studied to inform HWA control (Oten et al., 2014; Leppanen et al., 2019; Kinahan et al., 2020). Natural enemies are hypothesized to be at least partially responsible for controlling HWA populations in its native range (Cheah et al., 2004). However, hemlock species from the native range of the HWA (i.e., Asia) introduced to North America (e.g., *T. chinensis*, *T. dumosa*) support similarly low HWA populations in eastern North America where these same predators are absent (Bentz et al., 2002, 2007; Leppanen et al., 2019), suggesting a bottom-up resistance to HWA in some hemlock species. This apparent resistance may be conferred through differences in twig tissue chemistry (McKenzie et al., 2014) or cuticle thickness (Oten et al., 2012). Another possibility is that resistance to insect herbivory may, in part, originate from the plant microbiome, as has been demonstrated in some plants (e.g., Mejía et al., 2014; Garbelotto et al., 2019; Hubbard et al., 2019).

Initial investigations of the hemlock microbiome show that the branch microbiome varies across hemlock species, differing between HWA-susceptible and HWA-resistant species (Rogers et al., 2018). However, among HWA-susceptible hemlock species, the microbiome did not differ significantly between HWA population levels. Although these observations suggest that HWA infestation is independent of the plant microbiome, this initial work was limited in replication (*n* = 3) and investigated only the branch microbiome. It is also possible for outcomes of interactions with the microbiome associated with pest populations to appear in tissues away from the feeding site. For example, the soil microbiome can influence plant secondary metabolism impacting resistance to herbivory (Hubbard et al., 2019). Furthermore, even if the microbiome does not influence HWA populations, HWA feeding and subsequent associated damage still may affect the hemlock microbiome, e.g., because HWA infestation causes a plant immune response and the release of methyl salicylate into the vascular tissue (Pezet et al., 2013). In the rhizosphere of *Populus trichocarpa*, the concentration of salicylic acid correlated with the abundance of many bacterial and fungal phyla (Veach et al., 2019). Hence, a more systemic evaluation of the hemlock microbiome associated with HWA infestations and resistance is needed to reveal potentially important interactions.

To determine associations between the hemlock microbiome and HWA, we investigated the microbiome of two hemlock species, *T. canadensis* and *T. sieboldii*, with different HWA population levels across three plant tissue endospheres (e.g., needle, branch, and root) and their rhizosphere soils. Collectively, we use the term “plant-associated habitats” to describe the plant tissue endospheres and rhizosphere. *Tsuga canadensis* is native to eastern North America, and *T. sieboldii* is native to southern Japan (within the native range of HWA, Havill et al., 2016a) but has been introduced throughout the eastern USA (Farjon, 2010). We hypothesize that microbial α-diversity and community composition will differ among plant-associated habitats and between host species as has been shown previously in hemlock (Rogers et al., 2018) and other tree species such as *Populus* (Cregger et al., 2018), *Ginkgo* (Leff et al., 2015) and *Broussonetia* (Chen et al., 2020). However, we also hypothesize that microbial α-diversity and community composition will also differ across HWA population levels. We hypothesize that these differences in microbial community composition will correlate with changes in plant and soil chemistry associated with different host species and HWA population levels. We are also interested in the differences in specific microbial taxa with different hemlock host species and HWA population levels, specifically potential fungal pathogens and mycorrhizal fungi, which are well-characterized in the literature (Nguyen et al., 2016) and are known to affect plant host survivability (Smith and Read, 2008; Dean et al., 2012). We thus hypothesize that the relative abundance of potential fungal pathogens will increase with high HWA population levels due to compromised host defenses (Pezet et al., 2013). Additionally, the relative abundance of mycorrhizal fungi will decrease and the composition of mycorrhizal fungi will differ with high HWA population levels owing to altered resource allocation belowground with HWA infection (Gehring and Whitham, 1994a, b). Our overall goal is to describe the hemlock microbiome across plant tissues and host species and to identify microbial taxa associated with different HWA population levels that might subsequently be considered in HWA control.

## Materials and Methods

### Site Description and Sample Collection

Hemlock samples were collected on June 23, 2018, at the North Carolina State University Mountain Horticultural Crops Research Station, Mills River, NC, United States (35.420468 N, −82.556092 E, altitude: 643 m). Here, a variety of hemlock species were planted in a mixed-use forested landscape in 2008. Soils are characterized as Hayesville series (clayey, kaolinitic, mesic Typic Kanhapludults). For 2018 and 2019, mean annual precipitation was 176 cm, and temperature between October–March was 7.6°C and April–September was 19.6°C.

We collected samples from 40 hemlock trees across the two hemlock species (*T. canadensis* and *T. sieboldii*) and two HWA population levels (high and low) in full factorial design (10 replicate trees per species-HWA population level combination). Trees were characterized as having low HWA population levels when fewer than 10 HWA ovisacs were detected during 10 min censuses of the entire tree, and trees were characterized as having high HWA population levels when ovisacs were detected on >16 of 20 surveyed branches, five in each cardinal direction from approximately 0.1, 0.5, 1.0, 1.5, and 2.0 m above the ground. From each tree, we collected three 10 cm terminal branches from northeast, south–southeast, and west–northwest facing foliage at 1.5 m in height. These samples were composited and frozen on dry ice (−80°C) until sample pre-processing in the laboratory. We also collected fine roots (<2 mm diameter) and the attached soil, which we operationally defined as the rhizosphere, at each tree. Root and rhizosphere collections occurred in the upper 10 cm of soil and within 1 m of the base of the tree. All roots were traced back to the base of the tree. These samples were also frozen on dry ice until sample pre-processing.

### Sample Pre-processing and DNA Extraction

Prior to DNA extraction, needles, branches, and fine roots were washed and surface-sterilized as described by Cregger et al. (2018). To increase DNA yield prior to extraction, 50 mg of tissue per sample were cut into ∼5 mm pieces, flash-frozen in liquid nitrogen, and homogenized by bead-beating with a sterile 6 mm steel bead for two 1-minute intervals. Samples went through an additional flash freeze between intervals to prevent thawing. The DNA extractions were performed using the Qiagen PowerPlant Pro DNA Kit (Qiagen, Venlo, Netherlands) following the standard protocol with a slight procedural modification to ensure high-quality, high-concentration DNA yields. This modification consisted of homogenizing in a Precellys 24 (OMNI International, Kennesaw, Georgia, United States) at 3200 g for 3 min at 30 s intervals of pulse and rest. Rhizosphere soil was collected as the pre-sterilized rinsate of the fine roots. Rinsates were centrifuged at 10,000 rcf, and we removed the supernatant. We then used the Qiagen PowerSoil DNA Kit (Qiagen, Venlo, Netherlands) to extract these rinsates, following the standard protocol with the same modification to the procedure as seen above. Extractions were quantified on a NanoDrop 1000 spectrophotometer (NanoDrop Products, Wilmington, DE, United States). We used a Zymo DNA Clean and Concentrator-5 kit (Zymo Research Corporation, Irvine, CA, United States) to purify and concentrate needle, branch, and fine root endosphere extractions prior to polymerase chain reaction (PCR) amplification.

### PCR Amplification, Sequencing, and Bioinformatics

Archaeal/bacterial libraries were prepped for 16S rRNA gene sequencing by means of a two-step polymerase chain reaction (PCR) approach with a mixture of custom 515F and 806R primers (Cregger et al., 2018; Rogers et al., 2018), and for fungi using the ITS2 gene region with a custom mixture of primers (Cregger et al., 2018; Rogers et al., 2018; Supplementary Table S1). An adapter sequence was added to each forward and reverse primer to make them compatible with Nextera XT indexes (Illumina). The initial polymerase chain reaction (PCR) consisted of 2 × KAPA HiFi HotStart ReadyMix Taq (Roche, Indianapolis, Indiana, United States), 10 μmol/L total for each forward primer combination, and 10 μmol/L total for each reverse primer combination, with approximately 25 ng DNA. The 16S rRNA and ITS2 PCRs were performed separately. Both reactions consisted of 3 min at 95°C, followed by 25 cycles of 95°C for 30 s, 55°C for 30 s, and 72°C for 30 s, with a final extension at 72°C for 5 min. Successful PCR amplification was confirmed by running 4 μL of PCR product on a 2% agarose gel. The PCR product was then purified by use of AMPure XP beads (Agencourt, Beverly, MA, United States). Nextera XT indexes were then added to the PCR products by use of a second, reduced cycle PCR, such that each sample had a unique combination of forward and reverse indexes. This reduced reaction was the same as the previous reaction but with only eight cycles. The products were purified again using AMPure XP beads. Samples were quantified on a NanoDrop spectrophotometer (Fisher Scientific) and pooled into an archaeal/bacterial pool and a fungal pool to approximately equal concentrations within each pool. Final product sizes and concentrations were confirmed on an Agilent Bioanalyzer (Santa Clara, CA, United States) using the standard sensitivity kit. Both bacterial and fungal libraries were diluted to 4 μmol/L, independently combined with 5% of a 4 μmol/L PhiX adapter-ligated library control, and run paired-end on a v2, 500 cycle flow cell of an Illumina MiSeq sequencer.

Demultiplexed sequences were imported into the QIIME2 environment (Bolyen et al., 2019), and the median Phred quality scores of joined sequences were visualized. Both 16S and ITS2 datasets were denoised and classified into sequence variants (SVs) with the DADA2 algorithm in QIIME2 with reads truncated to 200 bases with the first 25 bases trimmed for 16S and reads truncated to 230 bases with the first 13 bases trimmed for ITS (Callahan et al., 2016). We then assigned representative sequences a taxonomic classification using Naïve Bayes classifier through the sklearn python package for 16S rRNA sequences with the SILVA database (Release 132; Quast et al., 2013), and we assigned taxonomic classifications to ITS rRNA representative sequences using BLAST and the UNITE reference database (version 8.0, Abarenkov et al., 2010). We removed contaminants (unassigned reads, mitochondria, chloroplasts for 16S; Protista, Chromista, Animalia, and Plantae reads for ITS2).

### Soil and Plant Chemical Analysis

To determine correlations between microbial community composition and plant and soil chemistry, branch, root, and rhizosphere samples were sent to the University of Georgia Extension Soil, Plant, and Water Laboratory for chemical analyses. Branch and root tissues as well as rhizosphere soils were ground and analyzed for total carbon (C) and nitrogen (N) concentrations by direct combustion using the Elementar vario MAX CNS Element Analyzer (Elementar, Langenselbold, Germany). Additionally, rhizosphere soils were analyzed for pH and lime buffer capacity (LBC). Briefly, pH was measured in a well-mixed 1:1_w:v_ soil:CaCl_2_ slurry (0.01 M) using a Fisherbrand accuTupH Rugged Double Junction pH Combination Electrode (Waltham, MA, United States). For LBC, pH was measured before, and 30 min after, a 2.7 ml addition of 0.023 M Ca(OH)_2_ to a 20 g soil and 20 ml 0.01 M CaCl_2_ slurry using the same pH electrode as above following Kissel et al. (2012).

### Statistical Analysis

All statistical analyses were conducted in R (R Development Core Team, 2008) with the phyloseq (McMurdie and Holmes, 2013), DESeq2 (Love et al., 2014), hillR (Li, 2018), nlme (Pinheiro et al., 2017), and vegan (Oksanen et al., 2019) packages.

Differences in α-diversity were compared by means of Hill numbers (Jost, 2006) of the point estimate of samples rarified to 1,000 reads (highest number of reads present across all samples) at orders of *q* = 0 and *q* = 1 (full rarefaction curves are presented in Supplementary Figure S1). The parameter q determines the relative weighting of rare species. At *q* = 0, all species are weighted equally (richness); at *q* = 1, species are weighted proportionally to their relative abundance (analogous to Shannon’s index). Differences in means of Hill numbers among plant-associated habitats, host species, and HWA population levels were assessed by nested ANOVA with tree identity as a random effect. Because we were primarily interested in a HWA population-level effect, where we found significant interactions between HWA population level and host species and/or plant-associated habitat, we performed individual ANOVAs and corrected the *p*-values using the Benjamini and Hochberg false discovery rate adjustment (Benjamini and Hochberg, 1995). The resulting ANOVAs did not include a random effect if plant-associated habitat was not a dependent variable because the resulting models would have had one sample per tree. Where independent variables were significant, we assessed multiple comparisons by Tukey’s test of honest significant differences. We used Q-Q plots and scale-location plots to inspect normality and homoscedasticity, respectively.

Differences in the community composition of the archaeal/bacterial and fungal microbiomes among plant-associated habitats, between hemlock host species, and across HWA population levels were assessed by nested distance-based redundancy analysis (dbRDA) constraining permutations within individual tree. We used the varpart() function in vegan (Oksanen et al., 2019) to determine the variance explained by each factor in our dbRDAs. For the dbRDAs, we used quantitative Jaccard (Ružička) distances applied to proportionally normalized data. Similar to our approach for α-diversity, where we found significant interactions, we performed individual dbRDAs for each host species or plant-associated habitat and corrected the *p*-values as described above. When performing separate dbRDAs for each plant-associated habitat, we similarly did not constrain permutations by tree because we had only one sample per tree.

We also assessed differences in community composition associated with HWA population level by identifying differentially abundant SVs across HWA population levels in each plant-associated habitat across host species. To do this, we first normalized the SV table through variance stabilization, then estimated the fold change of differentially abundant microbial SVs between low and high HWA population levels using Wald tests and shrinkage estimation for dispersions (Love et al., 2014) and similarly adjusted *p*-values of differentially abundant SVs with the Benjamini and Hochberg false discovery rate adjustment (Benjamini and Hochberg, 1995).

To assess the variation of the microbial community explained solely by plant and soil chemistry, we conducted dbRDAs for each plant-associated habitat individually (and therefore did not have to constrain by tree) using only the plant and soil variables as independent variables in the models. We conducted separate dbRDAs for each plant-associated habitat because we used only proximal plant chemical data (e.g., we did not attempt to correlate root C:N with the branch microbiome because we had chemical data from the tree branch). We determined the variation explained by each predictor with variance partitioning using the varpart() function in vegan (Oksanen et al., 2019).

Differences in fungal potential pathogen and mycorrhizal relative abundance (assessed by FUNGuild, Nguyen et al., 2016) among plant-associated habitats, host species, and HWA population levels were assessed by nested ANOVA and Tukey’s test of honest significant differences similarly to our approach for α-diversity. The resulting models were similarly inspected for normality and homoscedasticity. To satisfy these assumptions, the dependent variable in each model was log-transformed. We also assessed differences in the ectomycorrhizal community composition of the root and rhizosphere separately between host species and across HWA population levels using dbRDA, similarly using quantitative Jaccard (Ružička) distances applied to proportionally normalized data.

## Results

### Sequencing Results

After quality and taxonomic filtering (i.e., removal of plant and plasmid DNA), we sequenced 6.30 × 10^6^ 16S reads across 142 samples [18 samples were removed due to low read depths (<1,000)], with a minimum read depth of 1,448 and a maximum of 244,262. For ITS, we sequenced 5.92 × 10^6^ reads across 160 samples with a minimum read depth of 1,586 and a maximum of 122,712.

### Alpha Diversity

#### Archaeal/Bacterial Community

**FIGURE 1:**
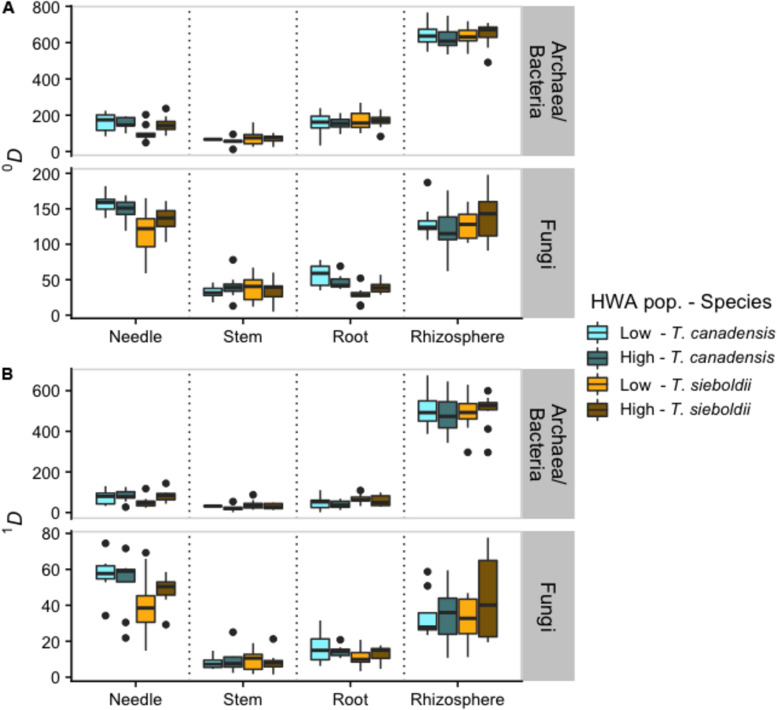
Boxplots representing α-diversity based on Hill numbers (Jost, 2006) of archaea/bacteria and fungi across plant-associated habitats, hemlock woolly adelgid (HWA) population levels, and host species at *q* = 0 (richness) **(A)** and *q* = 1 (analogous to Shannon diversity) **(B)**. Note different axis scales.

Archaeal/bacterial α-diversity (at *q* = 0 and 1) differed across plant-associated habitats (*q* = 0: *F*_3,88_ = 1047.358, *p* < 0.001; *q* = 1: *F*_3,88_ = 744.668, *p* < 0.001; Figure 1) with the microbiome of the rhizosphere being more diverse than the plant tissue microbiomes (all comparisons: *p* < 0.001). Also, at *q* = 0 (i.e., richness), the branch microbiome was less rich than the needle and root microbiomes (both comparisons: *p* < 0.01). We also detected greater archaeal/bacterial α-diversity in *T. canadensis* compared to *T. sieboldii*, but only at *q* = 0 (*q* = 0: *F*_1,37_ = 8.539, *p* = 0.006; *q* = 1: *F*_1,37_ = 1.482, *p* = 0.231). We failed to detect an effect of HWA population on archaeal/bacterial α-diversity (*q* = 0: *F*_1,37_ = 0.964, *p* = 0.333; *q* = 1: *F*_1,37_ = 0.759, *p* = 0.389).

#### Fungal Community

Plant-associated habitat and host species interacted in their effect on fungal α-diversity (*q* = 0: *F*_3,107_ = 6.031, *p* < 0.001; *q* = 1: *F*_3,107_ = 4.023, *p* = 0.009; Figure 1). Therefore, we analyzed the differences in fungal α-diversity for each plant-associated habitat individually. At *q* = 0, host species and HWA population level interacted in their effect on needle and root fungal richness (needle: *F*_1,36_ = 4.422, *p* = 0.043; root: *F*_1,36_ = 5.457, *p* = 0.025) such that diversity in *T. canadensis* exceeded that in *T. sieboldii* only at low HWA population levels (needle-low: *p* < 0.001, needle-high: *p* = 0.386, root-low: *p* < 0.001, root-high: *p* = 0.486). At q = 1, *T. canadensis* had greater needle and root fungal α-diversity compared to *T. sieboldii* regardless of HWA population level (needle: *F*_1,36_ = 7.505, *p* = 0.010; root: *F*_1,36_ = 3.906, *p* = 0.056). We detected no effect of host species or HWA population level on branch or rhizosphere fungal α-diversity at *q* = 0 and *q* = 1 (all: *p* > 0.1).

### Microbial Community Composition

#### Archaeal/Bacterial Community

Plant-associated habitat explained 21.3% of the variation in archaea/bacteria community composition (*p* < 0.001, adj-*R*^2^ = 0.203). Because there was a three-way interaction among plant-associated habitat, host species, and HWA population level (*p* = 0.068), we analyzed each plant-associated habitat separately.

**FIGURE 2:**
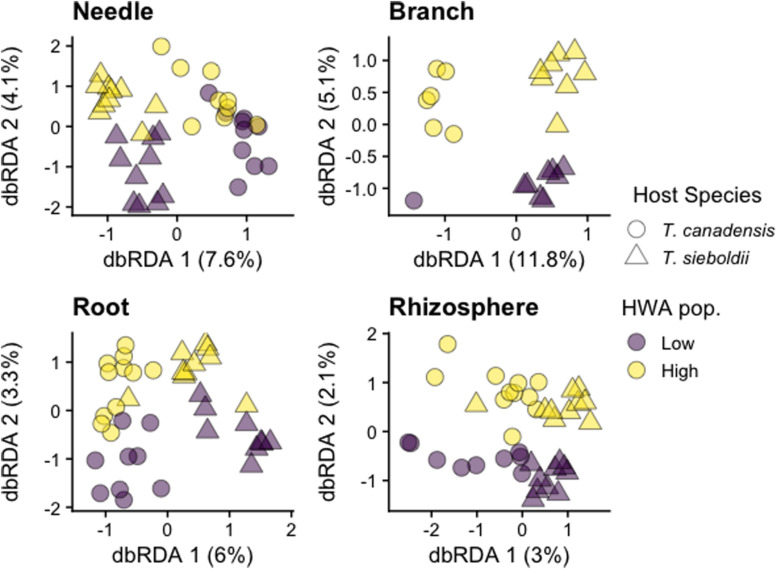
Distance-based redundancy analysis (dbRDA) ordinations of archaea/bacteria community composition across plant-associated habitats, hemlock woolly adelgid (HWA) population levels, and host species. Note different axis scales.

Hemlock woolly adelgid population level explained 2 and 1% of the variation in the needle and branch archaea/bacteria microbiomes, respectively, across both hemlock species (needle: *p* = 0.002, adj-*R*^2^ = 0.019; branch: *p* = 0.089, adj-*R*^2^ = 0.008; Figure 2). Additionally, there was a host species^∗^HWA population-level interaction in the root archaea/bacteria microbiome (*p* = 0.011) such that there was a greater HWA population-level effect in *T. sieboldii* (*p* = 0.036) than in *T. canadensis* (*p* = 0.052). At the phylum level, needles on trees with high HWA populations had greater abundance of Actinobacteria and lower abundance of Proteobacteria compared to needles on trees with low HWA populations, and the branch microbiome of trees with high HWA populations had greater abundance of Bacteroidetes and lower abundance of Actinobacteria compared to the branch microbiomes of trees with low HWA populations (Supplementary Figure S2). At the order level, high HWA population levels corresponded with high levels of Cytophagales in the needle microbiome and high levels of Betaproteobacteriales and Sphingomonadales in the branch microbiome (Supplementary Figure S3). For the needle, branch, and root archaea/bacteria microbiomes, there was a relatively stronger main effect of host species (needle: *p* < 0.001, adj-*R*^2^ = 0.048; branch: *p* < 0.001, adj-*R*^2^ = 0.074; root: *p* < 0.001, adj-*R*^2^ = 0.032), however, differences did not clearly emerge at the phylum level. Instead, these differences emerged at the family and genus level. For instance, we found that *T. sieboldii* had greater relative abundance of Beijerinckiaceae but a lower relative abundance of the genus *Candidatus Uzinura* (order: Flavobacteriales) in the branch microbiome compared to *T. canadensis* (Supplementary Figures S4, S5). We detected no effect of either HWA population level (*p* = 0.894) or host species (*p* = 0.182) on the rhizosphere archaea/bacteria microbiome composition. The effect of HWA population level on the microbial community also emerged at the sequence variant (SV) level. Hemlock woolly adelgid population level was associated with four differentially abundant archaeal and 1,057 differentially abundant bacterial SVs across the four plant-associated habitats (some SVs are shared among plant-associated habitats; needle: 104 SVs, 6.4% of SV richness; branch 8 SVs, 1.2% of SV richness; root: 173 SVs, 3.8% of SV richness; rhizosphere: 791 SVs, 2.7% of SV richness; Supplementary Figure S6 and Supplementary Table S2).

#### Fungal Community

Plant-associated habitat explained 13% of the variation in fungal community composition (*p* < 0.001). Because of a three-way interaction among plant-associated habitat, host species, and HWA population level on the fungal microbiome (*p* < 0.001), we also analyzed each plant-associated habitat separately.

**FIGURE 3:**
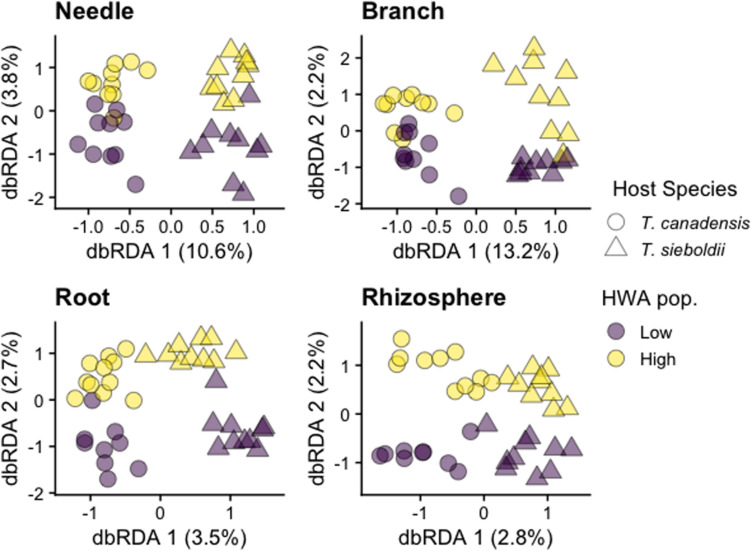
Distance-based redundancy analysis (dbRDA) ordinations of fungal community composition across plant-associated habitats, hemlock woolly adelgid (HWA) population levels, and host species. Note different axis scales.

Hemlock woolly adelgid population level explained 1% of the variation in the needle fungal community composition in both host species (*p* = 0.008, adj-*R*^2^ = 0.013, Figure 3). We detected no association between HWA population level and the composition of branch (*p* = 0.472), root (*p* = 0.174), and rhizosphere (*p* = 0.924) fungal communities (Figure 3). Except for the rhizosphere, composition of all other plant microbiomes was influenced by host species (needle: *p* < 0.001, *R*^2^ = 0.082; branch: *p* < 0.001, *R*^2^ = 0.109; root: *p* < 0.001, adj-*R*^2^ = 0.009; rhizosphere: *p* = 0.204). These differences in host species emerged at the class level with greater relative abundance of Teliomycetes (particularly order Helotiales) in the needles and branches and of Dothideomycetes (particularly order Pleosporales) in the roots of *T. canadensis* compared to those of *T. sieboldii* (Supplementary Figures S7, S8). Differences at the family and genus level were more nuanced because taxonomic classification at these levels is for the most part incomplete (Supplementary Figures S9, S10). Hemlock woolly adelgid population level was also associated with 1,481 differentially abundant fungal SVs across the four plant-associated habitats (some SVs are shared among plant-associated habitats; needle: 583 SVs, 19.1% of SV richness; branch: 168 SVs, 17.3% of SV richness; root: 298 SVs, 27.5% of SV richness; rhizosphere: 665 SVs, 19.5% of SV richness; Supplementary Figure S11 and Supplementary Table S3).

### Correlation of Microbiomes With Soil and Habitat Characteristics

**FIGURE 4:**
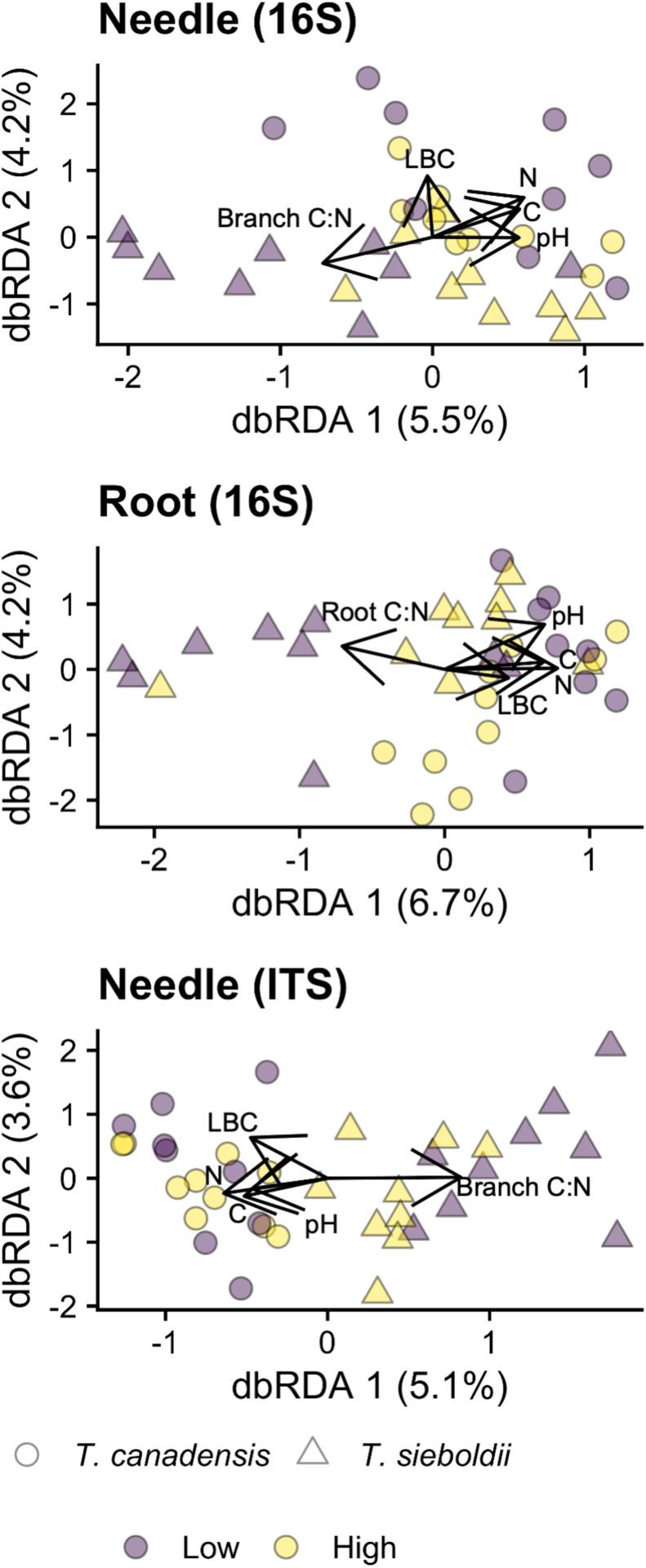
Distance-based redundancy analysis (dbRDA) ordinations of the needle archaeal/bacterial (16S) community composition, root archaeal/bacterial community composition, and needle fungal (ITS) community composition across hemlock woolly adelgid (HWA) population levels and host species. Microbial community compositions were ordinated along the variables soil total carbon (C), total nitrogen (N), pH (1:2 CaCl_2_), and lime buffering capacity LBC as well as the C:N of the plant-associated habitat (rhizosphere, root, or branch [same for needle]). Other environmental variable dbRDAs were not significant (*p* < 0.05). Environmental variables are represented by arrows, and bolded labels represent significant (*p* < 0.05) variables in the dbRDAs. Note different axis scales.

Total soil C, total soil N, branch C:N, root C:N, pH, and LBC were correlated with the composition of the needle archaeal/bacterial community (*p* = 0.006, variance explained: 17.3%), the root archaeal/bacterial community (*p* = 0.003, variance explained: 17.8%), and the needle fungal community (*p* = 0.046, variance explained: 15.7%; Figure 4). We detected no correlations between the plant and soil chemistry data and the microbial community for all other microbial community × plant-associated habitat combinations (*p* > 0.1). For the needle archaeal/bacterial community, lime buffer capacity (LBC) explained 1% of the variation in community composition (*p* = 0.014, adj-*R*^2^ = 0.011), and branch C:N explained 2% of the variation in community composition (*p* = 0.078, adj-*R*^2^ = 0.016). Root C:N and soil pH explained 2% and 3%, respectively, of the root archaeal/bacterial microbiome composition (root C:N: *p* = 0.012, adj-*R*^2^ = 0.025, pH: *p* = 0.026, adj-*R*^2^ = 0.020). Branch C:N and LBC explained 2 and 1%, respectively, of the needle fungal microbiome composition (Branch C:N: *p* = 0.072, adj-*R*^2^ = 0.015, LBC: *p* = 0.073, adj-*R*^2^ = 0.006).

### Fungal Potential Pathogens and Mycorrhizal Fungi

**FIGURE 5:**
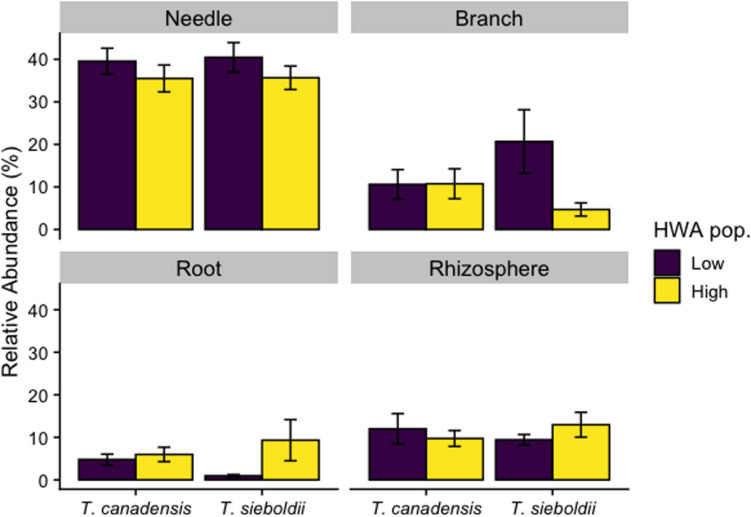
Relative abundance of fungal potential pathogen reads as a proportion of all fungal reads across plant-associated habitats, hemlock woolly adelgid (HWA) population levels, and host species.

One hundred fungal SVs classified as potential pathogens across the four plant-associated habitats were associated with high HWA populations, and about half of these were found in aboveground plant tissues (Supplementary Table S3). Analyzing the relative abundance of fungal potential pathogens in our samples, we detected a three-way interaction among plant-associated habitat, host species, and HWA population level (*F*_3,107_ = 2.801, *p* = 0.044, Figure 5). Therefore, we analyzed each plant-associated habitat separately. When analyzed separately for each plant-associated habitat, the relative abundance of fungal potential pathogens was comparable overall among HWA population levels and host species, except in specific instances. For example, there was an almost 10-fold greater relative abundance of potential pathogens in the roots of *T. sieboldii* with a high HWA population level compared to *T. sieboldii* with a low HWA population level (*p* = 0.029) (the relative abundance of potential pathogens in *T. canadensis* roots did not vary among HWA population levels [*p* = 0.988]). Between host species, the root fungal microbiome had a 5-fold greater relative abundance of potential pathogens in *T. canadensis* than in *T. sieboldii* only with low HWA population levels (*p* = 0.049).

**FIGURE 6:**
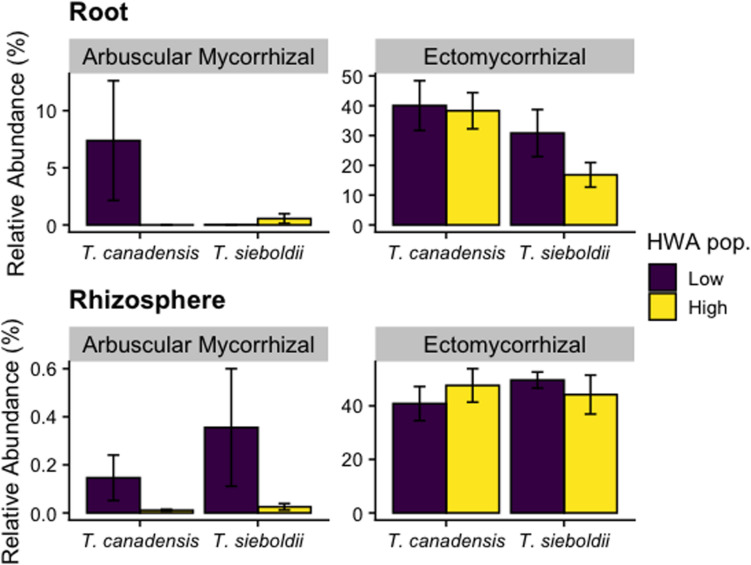
Relative abundance of arbuscular mycorrhizal (AM) and ectomycorrhizal (EM) fungal reads as a proportion of all fungal reads in the roots and rhizosphere across hemlock woolly adelgid (HWA) population levels and host species. Note different axis scales.

The relative abundance of ectomycorrhizal (EM) fungi in the root endosphere in *T. canadensis* significantly exceeded that in *T. sieboldii* (*p* = 0.025, Figure 6). However, there was no host species effect on the relative abundance of EM fungi in the rhizosphere (*p* = 0.693). Similarly, HWA population level was not associated with EM fungal relative abundance in the roots (*p* = 0.297) or rhizosphere (*p* = 0.930). The EM community composition did not vary between HWA population levels and host species in both roots (HWA: *p* = 0.736; host species: *p* = 0.281) and rhizosphere samples (HWA: *p* = 0.778; host species: *p* = 0.107; Supplementary Figure S12).

## Discussion

Consistent with our hypothesis, HWA population level was associated with many specific microbial taxa in the microbiomes of *T. canadensis* and *T. sieboldii* across multiple plant tissues and the rhizosphere at the SV level. Such findings, however, are inconsistent with previous research that found no association of HWA population levels with the branch microbiome (Rogers et al., 2018). By increasing the sample size compared to that of Rogers et al. (2018) (10 vs. 3), increasing the scope of the sampling to include other plant-associated habitats, and by investigating HWA-hemlock microbiome associations at multiple scales (e.g., SV level, community level), we were able to detect a significant relationship between HWA population level and the hemlock microbiome.

At the community-level, we detected a significant HWA population level association with the hemlock needle microbiome for both archaea/bacteria and fungi. It is not surprising that the needle microbiome had the strongest association with HWA population level because HWA infestation can affect nutrient delivery to the needles (Havill et al., 2016b). However, we found little effect of altered nutrient status on the needle microbiome in our environmental variable dbRDA, likely because we did not measure needle C or N and used branch C and N instead as a proxy. Future work should measure the nutrient content of the needles, including micronutrients, which may affect microbial community composition as well (Kembel et al., 2014), to test the hypothesis that HWA-induced changes in nutrient content affect the needle microbiome.

Infestation of HWA may also affect plant performance by increasing plant susceptibility to pathogens, either by compromising the plant defense system (e.g., Pezet et al., 2013) or by increasing labile substrate in the affected plant tissues (e.g., Tooker and De Moraes, 2009). Specifically, we found about an 8-fold enrichment of two *Gibberella* spp. SVs (which could not be classified to species resolution) in the needle microbiome of trees with high HWA populations (Supplementary Figure S11 and Supplementary Table S3). *Gibberella* species are globally widespread plant pathogens associated with many plant hosts, and they have multiple modes of pathogenesis (Desjardins, 2003). In an agricultural study, *Gibberella* ear rot severity in corn (*Zea mays*) was linked with the western bean cutworm (*Striacosta albicosta*) infestation (Parker et al., 2017), highlighting the interaction between plant pests and fungal pathogens. These *Gibberella* spp. SVs and the other 98 SVs classified as potential pathogens that were associated with high HWA population levels should be prioritized for future study of the interaction between HWA infestation and the hemlock microbiome.

We present preliminary evidence that hemlocks with high HWA population levels are selecting for microorganisms that may improve plant defense. For instance, we detected an eight-fold (on average) enrichment of two *Mycobacterium* and two *Pseudomonas* SVs (Supplementary Figure S6 and Supplementary Table S2) in the root endosphere; in some cases, these are known to produce salicylic acid (Ratledge and Winder, 1962; Visca et al., 1993; Lemanceau et al., 2017). Salicylic acid is an important plant defense compound (Pieterse et al., 2012), and by selecting for microorganisms with the capability to produce salicylic acid, plants may be better equipped to defend against pathogenesis (Lebeis et al., 2015). However, such evidence is highly speculative, and further metabolomic and transcriptomic work is necessary to determine if these taxa increase salicylic acid production in HWA-infested plants.

Lack of a mycorrhizal response to HWA population level is surprising in light of the fact that HWA not only reduces photosynthetic capacity (Nelson et al., 2014) and presumably C allocation belowground but also increases the nutrient supply in litter through increased throughfall (Stadler et al., 2006), both of which can decrease mycorrhizal colonization (Gehring and Whitham, 1994a, b). Resistance to HWA could be supported through mycorrhizal networks where mycorrhizae colonize multiple trees (Simard et al., 2012), altered growth strategies (e.g., mycorrhizal to saprotrophic, Johnson et al., 1997), or a delayed signal from the plant. Also, it was interesting that arbuscular mycorrhizal (AM) fungi were in such high abundance in hemlock roots, especially those of *T. canadensis* with low HWA population levels. Many of these AM fungi could not be identified beyond the family level (Glomeraceae). Hemlock species (family: Pinaceae) are not normally associated with AM fungi (Smith and Read, 2008), but in a greenhouse bioassay experiment, 25% of *T. heterophylla* seedlings were colonized by AM fungi (Cázares and Smith, 1995). Such findings counter the traditional paradigm that members of Pinaceae associate exclusively with EM fungi and promote the idea of mycorrhizal co-occurrence in Pinaceae (Wagg et al., 2008). Because the relative dominance of mycorrhizal types can potentially affect ecosystem-level processes (Phillips et al., 2013), the impact of HWA infestation on co-occurrence of AM and EM fungi in hemlock warrants detailed research.

Consistent with earlier work (Rogers et al., 2018), we found a greater percent of the variation in the microbiome composition explained by host species than by HWA population level. The effect of host species on the microbiome composition was strongest in the needles, branches, and roots, where the plant has a relatively stronger control over the microbiome environment (Kembel et al., 2014). Indeed, root C:N, which was, on average, about 12% lower in *T. canadensis* compared to *T. sieboldii*, was a significant determinant of microbiome composition across microbial domains. However, the differences in the microbiome composition among host species generally did not affect the relationship between the microbiome composition and HWA population level (i.e., no host species × HWA population level interaction). Therefore, we conclude that the microbiome compositions of these two HWA-susceptible species correlate with HWA population level in much the same way.

As with other studies (e.g., Beckers et al., 2017; Rossmann et al., 2017; Cregger et al., 2018), we found large differences in the composition of microbial communities among the different plant-associated habitats. Aboveground plant tissues were dominated by Alphaproteobacteria and Ascomycota, specifically two fungal classes: Dothideomycetes and Eurotiomycetes. Roots were dominated by Actinobacteria, and rhizospheres were enriched in Acidobacteria. Belowground habitats also had a greater proportion of Basidiomycota reads, specifically Agaricomycetes. These broad taxonomic patterns among different plant-associated habitats resemble those found in other temperate tree species such as *Magnolia kwangtungensis* (Qian et al., 2019), *Populus trichocarpa* and *P. deltoides* × *P. trichocarpa* hybrids (Cregger et al., 2018), and *Picea abies* (Kovalchuk et al., 2018; Ren et al., 2019; Terhonen et al., 2019), suggesting that, at higher taxonomic levels, microbiomes are fairly consistent among tree species. However, as our study and others show, at more specific taxonomic levels, microbiomes diverge among closely related host species (Cregger et al., 2018; Rogers et al., 2018) and even among different genotypes of the same host species (Bálint et al., 2013; Veach et al., 2019).

An important consideration of this work is that these results were obtained during a single sampling date. Indeed, microbiomes change seasonally and interannually (Redford and Fierer, 2009; Shade et al., 2013), and these temporal dynamics of the microbiome may increase or decrease our ability to distinguish ecological phenomena (Grady et al., 2019; Dove et al., 2020). Nevertheless, our results suggest modest to strong variations in the microbiome among HWA population levels, host species, and plant-associated habitats. Future work should determine the temporal robustness of these trends.

Investigating interactions among pests, microbial communities, and plant genetics contributes to a holistic understanding of the plant system that can be leveraged to promote plant health. Using 16S rRNA and ITS gene amplicon sequencing, we found a relatively modest relationship between HWA population level and the hemlock microbiome composition in two species. Nevertheless, even modest dissimilarities in the overall microbiome can result in functional consequences when specific driving taxa are differentially abundant (Agler et al., 2016). Future work should specifically investigate interactions between HWA infestation and the differentially abundant taxa highlighted in this study, especially those classified as potential plant pathogens.

## Author’s Note

This manuscript has been authored by UT-Battelle, LLC under Contract No. DE-AC05-00OR22725 with the United States Department of Energy.

## Data Availability Statement

The datasets presented in this study can be found in the Sequence Read Archive (https://www.ncbi.nlm.nih.gov/bioproject/622552) under project ID 622552. The SV, taxonomy, and sample data tables used in this analysis can be found in Supplementary Tables S4–S9, and the R code for all statistics and figures can be found at https://github.com/nicholascdove/Hemlock_microbiome.

## Author Contributions

MC, JF, CL, TJR, and DS designed the study. MC, AL, CL, TGR, TJR, and DS collected the sample. AL and TGR established and maintained the study site. VB, CL, and TJR processed the sample and analyzed the laboratory. ND led the data analysis. ND and MC wrote the manuscript with critical input from all coauthors. All authors contributed to the article and approved the submitted version.

## Conflict of Interest

The authors declare that the research was conducted in the absence of any commercial or financial relationships that could be construed as a potential conflict of interest.

## References

[B1] Abarenkov K. Nilsson R. H. Larsson K.-H. I. Alexander J. Eberhardt U. Erland S. (2010). The UNITE database for molecular identification of fungi – recent updates and future perspectives. *New Phytol.* 186 281–285. 10.1111/j.1469-8137.2009.03160.x 20409185

[B2] Agler M. T. Ruhe J. Kroll S. Morhenn C. Kim S.-T. Weigel D. (2016). Microbial hub taxa link host and abiotic factors to plant microbiome variation. *PLoS Biol.* 14:e1002352. 10.1371/journal.pbio.1002352 26788878 PMC4720289

[B3] Badri D. V. Zolla G. Bakker M. G. Manter D. K. Vivanco J. M. (2013). Potential impact of soil microbiomes on the leaf metabolome and on herbivore feeding behavior. *New Phytol.* 198 264–273. 10.1111/nph.12124 23347044

[B4] Bálint M. Tiffin P. Hallström B. O’Hara R. B. Olson M. S. Fankhauser J. D. (2013). Host genotype shapes the foliar fungal microbiome of balsam poplar (*Populus balsamifera*). *PLoS One* 8:e53987. 10.1371/journal.pone.0053987 23326555 PMC3543377

[B5] Beckers B. Op De Beeck M. Weyens N. Boerjan W. Vangronsveld J. (2017). Structural variability and niche differentiation in the rhizosphere and endosphere bacterial microbiome of field-grown poplar trees. *Microbiome* 5:25. 10.1186/s40168-017-0241-2 PMC5324219 28231859

[B6] Benjamini Y. Hochberg Y. (1995). Controlling the false discovery rate: a practical and powerful approach to multiple testing. *J. R. Statist. Soc. Ser. B (Methodol.)* 57 289–300. 10.1111/j.2517-6161.1995.tb02031.x

[B7] Bentz S. E. Griesbach R. J. Pooler M. R. Townsend A. M. (2007). “*Tsuga chinensis* as a source of host resistance to the hemlock woolly adelgid,” in *Proceedings of the 17th U.S. Department of Agriculture Interagency Research Forum on Gypsy Moth and Other Invasive Species 2006; Gen. Tech. Rep. NRS-P-10*, Vol. 10 ed. Gottschalk K. W. (Newtown Square, PA: U.S. Department of Agriculture, Forest Service, Northern Research Station), 24–25.

[B8] Bentz S. E. Riedel L. G. H. Pooler M. R. Townsend A. M. (2002). Hybridization and self-compatibility in controlled pollinations of eastern North American and Asian hemlock (*Tsuga*) species.–Abstract–Europe PMC. *J. Arboricul.* 28 200–205.

[B9] Bolyen E. Rideout J. R. Dillon M. R. Bokulich N. A. Abnet C. C. Al-Ghalith G. A. (2019). Reproducible, interactive, scalable and extensible microbiome data science using QIIME 2. *Nat. Biotechnol.* 37 852–857. 31341288 10.1038/s41587-019-0209-9 PMC7015180

[B10] Busby P. E. Crutsinger G. Barbour M. Newcombe G. (2019). Contingency rules for pathogen competition and antagonism in a genetically based, plant defense hierarchy. *Ecol. Evol.* 9 6860–6868. 10.1002/ece3.5253 31380021 PMC6662256

[B11] Busby P. E. Peay K. G. Newcombe G. (2016). Common foliar fungi of *Populus trichocarpa* modify *Melampsora* rust disease severity. *New Phytol.* 209 1681–1692. 10.1111/nph.13742 26565565

[B12] Busby P. E. Zimmerman N. Weston D. J. Jawdy S. S. Houbraken J. Newcombe G. (2013). Leaf endophytes and *Populus* genotype affect severity of damage from the necrotrophic leaf pathogen, *Drepanopeziza populi*. *Ecosphere* 4:art125 10.1890/es13-00127.1

[B13] Callahan B. J. McMurdie P. J. Rosen M. J. Han A. W. Johnson A. J. A. Holmes S. P. (2016). DADA2: high-resolution sample inference from Illumina amplicon data. *Nat. Methods* 13 581–583. 10.1038/nmeth.3869 27214047 PMC4927377

[B14] Cázares E. Smith J. E. (1995). Occurrence of vesicular-arbuscular mycorrhizae in *Pseudotsuga menziesii* and *Tsuga heterophylla* seedlings grown in Oregon coast range soils. *Mycorrhiza* 6 65–67. 10.1007/s005720050108

[B15] Cheah C. Montgomery M. E. Salom S. Parker B. L. Costa S. Skinner M. (2004). *Biological Control of Hemlock Woolly Adelgid*, Vol. 28 Morgantown, WV: USDA, Forest Service, Forest Health Technology Enterprise Team.

[B16] Chen P. Zhao M. Tang F. Hu Y. Peng X. Shen S. (2020). The effect of plant compartments on the *Broussonetia papyrifera*-associated fungal and bacterial communities. *Appl. Microbiol. Biotechnol.* 104 3627–3641. 10.1007/s00253-020-10466-6 32078018

[B17] Compant S. Duffy B. Nowak J. Clément C. Barka E. A. (2005). Use of plant growth-promoting bacteria for biocontrol of plant diseases: principles, mechanisms of action, and future prospects. *Appl. Environ. Microbiol.* 71 4951–4959. 10.1128/aem.71.9.4951-4959.2005 16151072 PMC1214602

[B18] Cregger M. A. Veach A. M. Yang Z. K. Crouch M. J. Vilgalys R. Tuskan G. A. (2018). The *Populus* holobiont: dissecting the effects of plant niches and genotype on the microbiome. *Microbiome* 6:31. 10.1186/s40168-018-0413-8 PMC5810025 29433554

[B19] Dean R. Kan J. A. L. V. Pretorius Z. A. Hammond−Kosack K. E. Pietro A. D. Spanu P. D. (2012). The top 10 fungal pathogens in molecular plant pathology. *Mol. Plant Pathol.* 13 414–430. 10.1111/j.1364-3703.2011.00783.x 22471698 PMC6638784

[B20] Desjardins A. E. (2003). *Gibberella* from A (venaceae) to Z (eae). *Ann. Rev. Phytopathol.* 41 177–198. 10.1146/annurev.phyto.41.011703.115501 12651961

[B21] Dove N. C. Safford H. D. Bohlman G. N. Estes B. L. Hart S. C. (2020). High-severity wildfire leads to multi-decadal impacts on soil biogeochemistry in mixed-conifer forests. *Ecol. Appl.* 30:e02072. 10.1002/eap.2072 31925848

[B22] Ellison A. M. Bank M. S. Clinton B. D. Colburn E. A. Elliott K. Ford C. R. (2005). Loss of foundation species: consequences for the structure and dynamics of forested ecosystems. *Front. Ecol. Environ.* 3 479–486. 10.1890/1540-9295(2005)003[0479:lofscf]2.0.co;2

[B23] Eschtruth A. K. Evans R. A. Battles J. J. (2013). Patterns and predictors of survival in *Tsuga canadensis* populations infested by the exotic pest *Adelges tsugae*: 20 years of monitoring. *Forest Ecol. Manag.* 305 195–203. 10.1016/j.foreco.2013.05.047

[B24] Farjon A. (2010). *A Handbook of the World’s Conifers (2 vols.): Revised and Updated Edition.* Leiden: Brill.

[B25] Garbelotto M. Lowell N. I. Chen Y. Osmundson T. W. (2019). Evidence for inhibition of a fungal biocontrol agent by a plant microbiome. *J. Plant Pathol.* 101 457–466. 10.1007/s42161-019-00247-0

[B26] Gehring C. A. Whitham T. G. (1994a). Comparisons of ectomycorrhizae on pinyon pines (*Pinus edulis*; Pinaceae) across extremes of soil type and herbivory. *Am. J. Bot.* 81 1509–1516. 10.1002/j.1537-2197.1994.tb11461.x

[B27] Gehring C. A. Whitham T. G. (1994b). Interactions between aboveground herbivores and the mycorrhizal mutualists of plants. *Trends Ecol. Evol.* 9 251–255. 10.1016/0169-5347(94)90290-9 21236843

[B28] Grady K. L. Sorensen J. W. Stopnisek N. Guittar J. Shade A. (2019). Assembly and seasonality of core phyllosphere microbiota on perennial biofuel crops. *Nat. Commun.* 10 1–10. 31515535 10.1038/s41467-019-11974-4 PMC6742659

[B29] Havill N. P. Shiyake S. Galloway A. L. Foottit R. G. Yu G. Paradis A. (2016a). Ancient and modern colonization of North America by hemlock woolly adelgid, *Adelges tsugae* (Hemiptera: Adelgidae), an invasive insect from East Asia. *Mol. Ecol.* 25 2065–2080. 10.1111/mec.13589 26880353

[B30] Havill N. P. Viera L. C. Salom S. M. (2016b). *FHET-2014-05. Biology and Control of the Hemlock Woolly Adelgid.* Morgantown, WV: USDA Forest Service, 1–21.

[B31] Hubbard C. J. Li B. McMinn R. Brock M. T. Maignien L. Ewers B. E. (2019). The effect of rhizosphere microbes outweighs host plant genetics in reducing insect herbivory. *Mol. Ecol.* 28 1801–1811. 10.1111/mec.14989 30582660

[B32] Jenkins J. C. Aber J. D. Canham C. D. (1999). Hemlock woolly adelgid impacts on community structure and N cycling rates in eastern hemlock forests. *Canad. J. Forest Res.* 29 630–645. 10.1139/x99-034

[B33] Johnson N. C. Graham J.-H. Smith F. A. (1997). Functioning of mycorrhizal associations along the mutualism–parasitism continuum. *New Phytol.* 135 575–585. 10.1046/j.1469-8137.1997.00729.x

[B34] Jost L. (2006). Entropy and diversity. *Oikos* 113 363–375.

[B35] Kantola T. Tracy J. L. Lyytikäinen-Saarenmaa P. Saarenmaa H. Coulson R. N. Trabucco A. (2019). Hemlock woolly adelgid niche models from the invasive eastern North American range with projections to native ranges and future climates. *iForest Biogeosci. Forestry* 12:149. 10.3832/ifor2883-012 17959540

[B36] Karban R. Adamchak R. Schnathorst W. C. (1987). Induced resistance and interspecific competition between spider mites and a vascular wilt fungus. *Science* 235 678–680. 10.1126/science.235.4789.678 17833628

[B37] Kembel S. W. O’Connor T. K. Arnold H. K. Hubbell S. P. Wright S. J. Green J. L. (2014). Relationships between phyllosphere bacterial communities and plant functional traits in a neotropical forest. *Proc. Natl. Acad. Sci. U.S.A.* 111 13715–13720. 10.1073/pnas.1216057111 25225376 PMC4183302

[B38] Kinahan I. G. Grandstaff G. Russell A. Rigsby C. M. Casagrande R. A. Preisser E. L. (2020). A four-year, seven-state reforestation trial with eastern hemlocks (*Tsuga canadensis*) resistant to hemlock woolly adelgid (*Adelges tsugae*). *Forests* 11:312 10.3390/f11030312

[B39] Kissel D. E. Sonon L. S. Cabrera M. L. (2012). Rapid measurement of soil pH buffering capacity. *Soil Sci. Soc. Am. J.* 76 694–699. 10.2136/sssaj2011.0091

[B40] Kovalchuk A. Mukrimin M. Zeng Z. Raffaello T. Liu M. Kasanen R. (2018). Mycobiome analysis of asymptomatic and symptomatic Norway spruce trees naturally infected by the conifer pathogens *Heterobasidion* spp. *Environ. Microbiol. Rep.* 10 532–541. 10.1111/1758-2229.12654 29727054

[B41] Lebeis S. L. Paredes S. H. Lundberg D. S. Breakfield N. Gehring J. McDonald M. (2015). Salicylic acid modulates colonization of the root microbiome by specific bacterial taxa. *Science* 349 860–864. 10.1126/science.aaa8764 26184915

[B42] Leff J. W. Tredici P. D. Friedman W. E. Fierer N. (2015). Spatial structuring of bacterial communities within individual *Ginkgo biloba* trees. *Environ. Microbiol.* 17 2352–2361. 10.1111/1462-2920.12695 25367625

[B43] Lemanceau P. Blouin M. Muller D. Moënne-Loccoz Y. (2017). Let the core microbiota be functional. *Trends Plant Sci.* 22 583–595. 10.1016/j.tplants.2017.04.008 28549621

[B44] Leppanen C. Fordyce J. A. LeBude A. V. Ranney T. G. Simberloff D. (2019). Variable colonization by the hemlock woolly adelgid suggests infestation is associated with hemlock host species. *Biol. Invas.* 21 2891–2906. 10.1007/s10530-019-02020-x

[B45] Li D. (2018). hillR: taxonomic, functional, and phylogenetic diversity and similarity through Hill Numbers. *J. Open Source Softw.* 3:1041 10.21105/joss.01041

[B46] Love M. I. Huber W. Anders S. (2014). Moderated estimation of fold change and dispersion for RNA-seq data with DESeq2. *Genome Biol.* 15:550. 10.1186/s13059-014-0550-8 PMC4302049 25516281

[B47] Marcelino J. Giordano R. Gouli S. Gouli V. Parker B. L. Skinner M. (2008). C*olletotrichum acutatum* var. fioriniae (teleomorph: *Glomerella acutata* var. fioriniae var. nov.) infection of a scale insect. *Mycologia* 100 353–374. 10.3852/07-174r 18751543

[B48] McClure M. S. (1987). Biology and control of hemlock woolly adelgid. *Connect. Agricult. Exp. Stat. Bull.* 851 1–9.

[B49] McClure M. S. Salom S. M. Shields K. S. (1996). *Hemlock Woolly Adelgid.* Margan down, WV: USDA, Forest Service, Forest Health Technology Enterprise Team.

[B50] McKenzie E. A. Elkinton J. S. Casagrande R. A. Preisser E. I. Mayer M. (2014). Terpene chemistry of eastern hemlocks resistant to hemlock woolly adelgid. *J. Chem. Ecol.* 40 1003–1012. 10.1007/s10886-014-0495-0 25278447

[B51] McMurdie P. J. Holmes S. (2013). phyloseq: an R package for reproducible interactive analysis and graphics of microbiome census data. *PLoS One* 8:e61217. 10.1371/journal.pone.0061217 23630581 PMC3632530

[B52] Mejía L. C. Herre E. A. Sparks J. P. Winter K. García M. N. Bael S. A. Van, et al. (2014). Pervasive effects of a dominant foliar endophytic fungus on host genetic and phenotypic expression in a tropical tree. *Front. Microbiol.* 5:479. 10.3389/fmicb.2014.00479 25309519 PMC4162356

[B53] Muvea A. M. Meyhöfer R. Subramanian S. Poehling H.-M. Ekesi S. Maniania N. K. (2014). Colonization of onions by endophytic fungi and their impacts on the biology of *Thrips tabaci*. *PLoS One* 9:e108242. 10.1371/journal.pone.0108242 25254657 PMC4177896

[B54] Nelson L. A. Dillaway D. N. Rieske L. K. (2014). Effect of an exotic herbivore, Adelgestsugae, on photosynthesis of a highly susceptible *Tsuga* host, with notes on conspecifics. *Arthro. Plant Interact.* 8 9–15. 10.1007/s11829-013-9285-9

[B55] Nguyen N. H. Song Z. Bates S. T. Branco S. Tedersoo L. Menke J. (2016). FUNGuild: an open annotation tool for parsing fungal community datasets by ecological guild. *Fungal Ecol.* 20 241–248. 10.1016/j.funeco.2015.06.006

[B56] Oksanen J. Blanchet F. G. Kindt R. Legendre P. Simpson G. L. Minchin P. R. (2019). *vegan: Community Ecology Package.* Available online at: https://cran.r-project.org, https://github.com/vegandevs/vegan (accessed September 1, 2019).

[B57] Oten K. L. F. (2011). *Host-Plant Selection by the Hemlock Woolly Adelgid, Adelges tsugae Annand: Sensory Systems and Feeding Behavior in Relation to Physical and Chemical Host-Plant Characteristics.* Ph.D. dissertations, Department of Entomology, North Carolina State University, Raleigh, NC.

[B58] Oten K. L. F. Bauchan G. R. Frampton J. Hain F. P. (2012). Biophysical characteristics of the stem and petiole surface of six hemlock (*Tsuga*) species and a hybrid: implications for resistance to *Adelges tsugae*. *Botany* 90 1170–1178. 10.1139/b2012-095

[B59] Oten K. L. F. Merkle S. A. Jetton R. M. Smith B. C. Talley M. E. Hain F. P. (2014). Understanding and developing resistance in hemlocks to the hemlock woolly adelgid. *South. Natur.* 13 147–167.

[B60] Parker N. S. Anderson N. R. Richmond D. S. Long E. Y. Wise K. A. Krupke C. H. (2017). Larval western bean cutworm feeding damage encourages the development of *Gibberella* ear rot on field corn. *Pest Manag. Sci.* 73 546–553. 10.1002/ps.4313 27158946

[B61] Pezet J. Elkinton J. Gomez S. Mckenzie E. A. Lavine M. Preisser E. (2013). Hemlock woolly adelgid and elongate hemlock scale induce changes in foliar and twig volatiles of eastern hemlock. *J. Chem. Ecol.* 39 1090–1100. 10.1007/s10886-013-0300-5 23900803

[B62] Phillips R. P. Brzostek E. Midgley M. G. (2013). The mycorrhizal-associated nutrient economy: a new framework for predicting carbon–nutrient couplings in temperate forests. *New Phytol.* 199 41–51. 10.1111/nph.12221 23713553

[B63] Pieterse C. M. J. Van der Does D. Zamioudis C. Leon-Reyes A. Van Wees S. C. M. (2012). Hormonal modulation of plant immunity. *Ann. Rev. Cell Dev. Biol.* 28 489–521. 22559264 10.1146/annurev-cellbio-092910-154055

[B64] Pineda A. Kaplan I. Bezemer T. M. (2017). Steering soil microbiomes to suppress aboveground insect pests. *Trends Plant Sci.* 22 770–778. 10.1016/j.tplants.2017.07.002 28757147

[B65] Pinheiro J. Bates D. DebRoy S. Sarkar D. (2017). nlme: linear and nonlinear mixed effects models. *R Pack. Vers.* 3 1–117.

[B66] Qian X. Li H. Wang Y. Wu B. Wu M. Chen L. (2019). Leaf and root endospheres harbor lower fungal diversity and less complex fungal co-occurrence patterns than rhizosphere. *Front. Microbiol.* 10:1015. 10.3389/fmicb.2019.01015 31143169 PMC6521803

[B67] Quast C. Pruesse E. Yilmaz P. Gerken J. Schweer T. Yarza P. (2013). The SILVA ribosomal RNA gene database project: improved data processing and web-based tools. *Nucleic Acids Res.* 41 D590–D596. 23193283 10.1093/nar/gks1219 PMC3531112

[B68] R Development Core Team (2008). *R: A Language and Environment for Statistical Computing.* Vienna: R Foundation for Statistical Computing.

[B69] Ratledge C. Winder F. G. (1962). The accumulation of salicylic acid by mycobacteria during growth on an iron-deficient medium. *Biochem. J.* 84 501–506. 10.1042/bj0840501 14490535 PMC1243704

[B70] Redford A. J. Fierer N. (2009). Bacterial succession on the leaf surface: a novel system for studying successional dynamics. *Microb. Ecol.* 58 189–198. 10.1007/s00248-009-9495-y 19221834

[B71] Ren F. Kovalchuk A. Mukrimin M. Liu M. Zeng Z. Ghimire R. P. (2019). Tissue microbiome of Norway spruce affected by *Heterobasidion*-induced wood decay. *Microb. Ecol.* 77 640–650. 10.1007/s00248-018-1240-y 30094615

[B72] Rogers T. J. Leppanen C. Brown V. Fordyce J. A. LeBude A. Ranney T. (2018). Exploring variation in phyllosphere microbial communities across four hemlock species. *Ecosphere* 9:e02524 10.1002/ecs2.2524

[B73] Rossmann M. Sarango-Flores S. W. Chiaramonte J. B. Kmit M. C. P. Mendes R. (2017). “Plant microbiome: composition and functions in plant compartments,” in *The Brazilian Microbiome: Current Status and Perspectives*, eds Pylro V. Roesch L. (Cham: Springer International Publishing), 7–20. 10.1007/978-3-319-59997-7_2

[B74] Santoyo G. Moreno-Hagelsieb G. del Carmen Orozco-Mosqueda M. A. Glick B. R. (2016). Plant growth-promoting bacterial endophytes. *Microbiol. Res.* 183 92–99. 26805622 10.1016/j.micres.2015.11.008

[B75] Shade A. Gregory Caporaso J. Handelsman J. Knight R. Fierer N. (2013). A meta-analysis of changes in bacterial and archaeal communities with time. *SME J.* 7 1493–1506. 10.1038/ismej.2013.54 23575374 PMC3721121

[B76] Silcox C. A. (2002). *Using Imidacloprid to Control Hemlock Woolly Adelgid. Proceedings Hemlock Woolly Adelgid in the Eastern United States.* New Brunswick, NJ: NJ Agricultural Experiment Station, 280–287.

[B77] Simard S. W. Beiler K. J. Bingham M. A. Deslippe J. R. Philip L. J. Teste F. P. (2012). Mycorrhizal networks: Mechanisms, ecology and modelling. *Fung. Biol. Rev.* 26 39–60. 10.1016/j.fbr.2012.01.001

[B78] Smith S. E. Read D. J. (2008). *Mycorrhizal Symbiosis*, 3rd Edn Cambridge, MA: Academic Press.

[B79] Snyder C. D. Young J. A. Lemarié D. P. Smith D. R. (2002). Influence of eastern hemlock (*Tsuga canadensis*) forests on aquatic invertebrate assemblages in headwater streams. *Canad. J. Fish. Aquat. Sci.* 59 262–275. 10.1139/f02-003

[B80] Stadler B. Müller T. Orwig D. (2006). The ecology of energy and nutrient fluxes in hemlock forests invaded by hemlock woolly adelgid. *Ecology* 87 1792–1804. 10.1890/0012-9658(2006)87[1792:teoean]2.0.co;2 16922328

[B81] Terhonen E. Blumenstein K. Kovalchuk A. Asiegbu F. O. (2019). Forest tree microbiomes and associated fungal endophytes: functional roles and impact on forest health. *Forests* 10:42 10.3390/f10010042

[B82] Thaler J. S. Agrawal A. A. Halitschke R. (2010). Salicylate-mediated interactions between pathogens and herbivores. *Ecology* 91, 1075–1082. 10.1890/08-2347.1 20462121

[B83] Tibbets T. M. Faeth S. H. (1999). *Neotyphodium* endophytes in grasses: deterrents or promoters of herbivory by leaf-cutting ants? *Oecologia* 118 297–305. 10.1007/s004420050730 28307273

[B84] Tooker J. F. De Moraes C. M. (2009). A gall-inducing caterpillar species increases essential fatty acid content of its host plant without concomitant increases in phytohormone levels. *Mol. Plant Microb. Interact.* 22 551–559. 10.1094/mpmi-22-5-0551 19348573

[B85] Veach A. M. Morris R. Yip D. Z. Yang Z. K. Engle N. L. Cregger M. A. (2019). Rhizosphere microbiomes diverge among *Populus trichocarpa* plant-host genotypes and chemotypes, but it depends on soil origin. *Microbiome* 7:76. 10.1186/s40168-019-0668-8 PMC6525979 31103040

[B86] Visca P. Ciervo A. Sanfilippo V. Orsi N. (1993). Iron-regulated salicylate synthesis by *Pseudomonas* spp. *Microbiology* 139 1995–2001. 10.1099/00221287-139-9-1995 7504066

[B87] Wagg C. Pautler M. Massicotte H. B. Peterson R. L. (2008). The co-occurrence of ectomycorrhizal, arbuscular mycorrhizal, and dark septate fungi in seedlings of four members of the Pinaceae. *Mycorrhiza* 18 103–110. 10.1007/s00572-007-0157-y 18157555

[B88] Yamasaki M. DeGraaf R. M. Lanier J. W. (2000). “Wildlife habitat associations in eastern hemlock–birds, smaller mammals, and forest carnivores,” in *Proceedings: Symposium on Sustainable Management of Hemlock Ecosystems in Eastern North America. Gen. Tech. Rep. NE-267*, Vol. 267 eds McManus K. A. Shields K. S. Souto D. R. (Newtown Square, PA: U.S. Department of Agriculture, Forest Service, Northeastern Forest Experiment Station), 135–143.

[B89] Young R. F. Shields K. S. Berlyn G. P. (1995). Hemlock woolly adelgid (Homoptera: Adelgidae): stylet bundle insertion and feeding sites. *Ann. Entomol. Soc. Am.* 88 827–835. 10.1093/aesa/88.6.827

