# Supplementary material for: Microbiome Variation Across Two Hemlock Species With Hemlock Woolly Adelgid Infestation
Source: Front Microbiol. 2020 Jul 7;11:1528. doi: 10.3389/fmicb.2020.01528 (PMC7358439; doi:10.3389/fmicb.2020.01528)

**Supplemental Figures**

Microbiome variation in two hemlock species infested with hemlock woolly adelgid

Nicholas C. Dove^1^, Timothy J. Rogers^2^, Christy Leppanen^3^, Daniel Simberloff^3^, James A. Fordyce^3^, Veronica Brown^3^, Anthony V. LeBude^4^, Thomas Ranney^4^, and Melissa A. Cregger^1,3*^

^1^Biosciences Division, Oak Ridge National Laboratory, Oak Ridge, TN 37831, USA

^2^University of Tennessee, Department of Microbiology, Knoxville, Tennessee 37996 USA

^3^University of Tennessee, Department of Ecology and Evolutionary Biology, Knoxville, Tennessee 37996 USA

^4^North Carolina State University, Department of Horticultural Science, Mills River, North Carolina 28759 USA

*Corresponding Author: creggerma@ornl.gov

*This manuscript has been authored by UT-Battelle, LLC under Contract No. DE-AC05-00OR22725 with the U.S. Department of Energy. The United States Government retains and the*

*publisher, by accepting the article for publication, acknowledges that the United States Government retains a non-exclusive, paid-up, irrevocable, world-wide license to publish or reproduce the published form of this manuscript, or allow others to do so, for United States Government purposes. The Department of Energy will provide public access to these results of federally sponsored research in accordance with the DOE Public Access Plan (*[*http://energy.gov/downloads/doe-public-access-plan*](http://energy.gov/downloads/doe-public-access-plan)*).*

**Figure S1:** Rarefaction curves for 16S (A) and ITS (B) across plant-associated habitats, host species, and hemlock woolly adelgid (HWA) population levels.

**
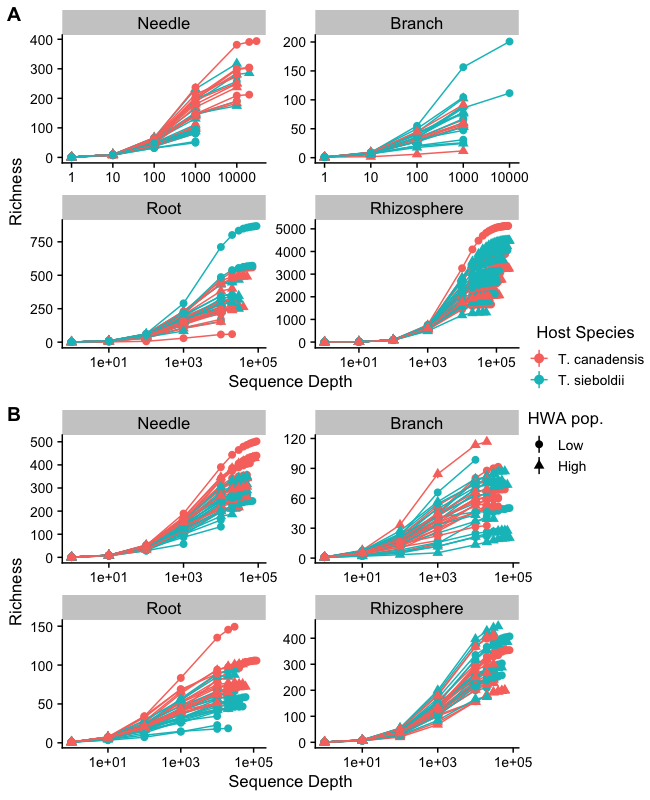
**

**Figure S2**: Relative read abundance of archaea and major bacterial taxonomic groups across plant-associated habitats, hemlock woolly adelgid population levels, and host species.


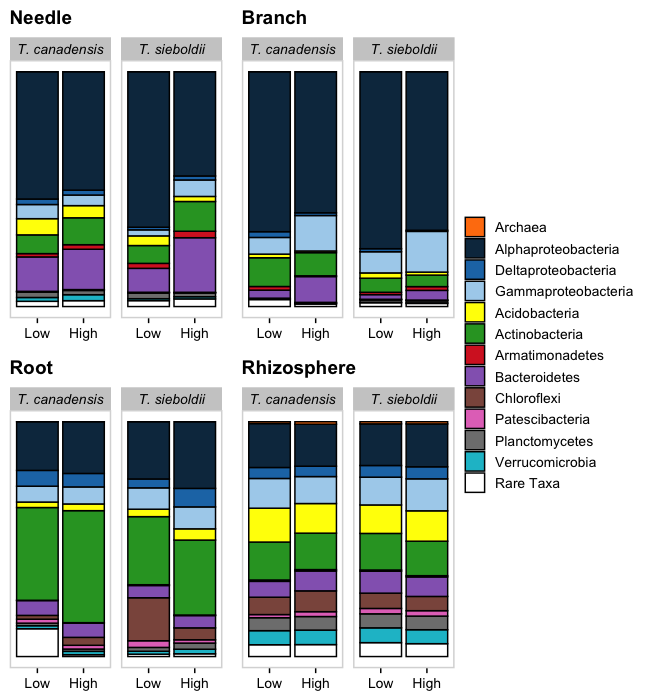


**Figure S3:** Relative read abundance of dominant bacterial orders across plant-associated habitats, hemlock woolly adelgid population levels, and host species.

**
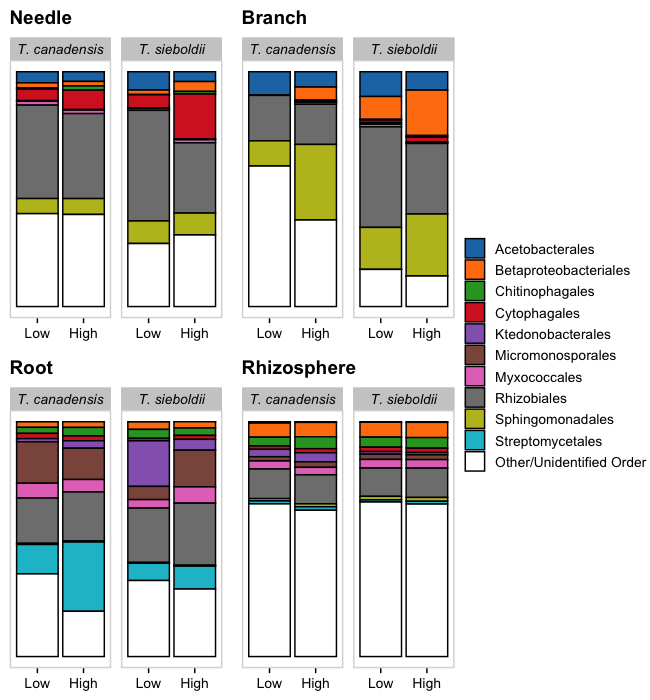
**

**Figure S4:** Relative read abundance of dominant bacterial families across plant-associated habitats, hemlock woolly adelgid population levels, and host species.

**
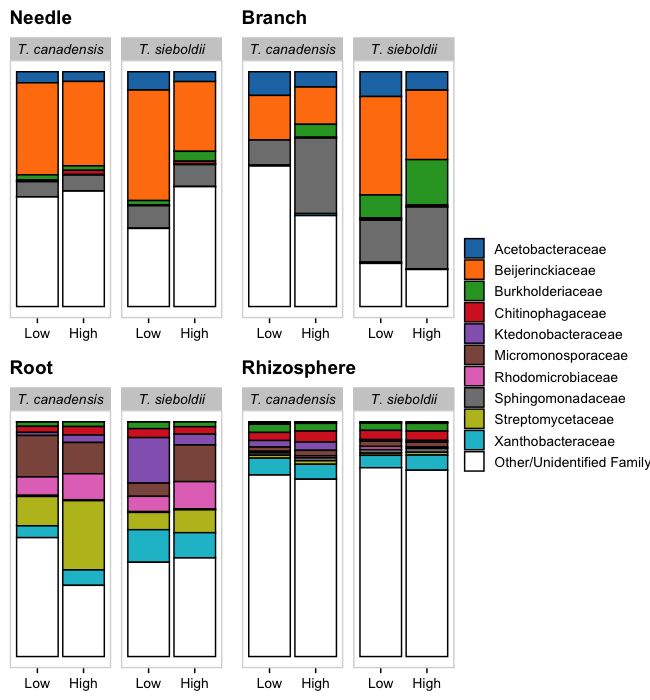
**

**Figure S5:** Relative read abundance of dominant bacterial genera across plant-associated habitats, hemlock woolly adelgid population levels, and host species.

**
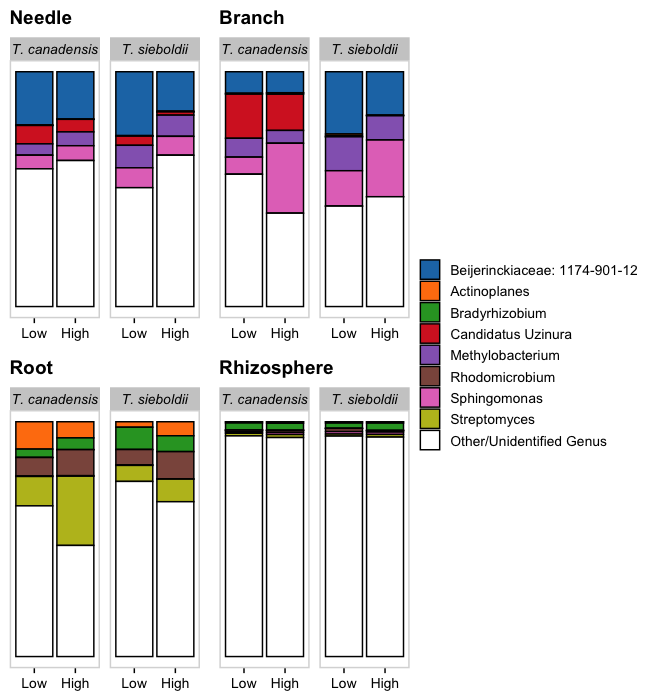
**

**Figure S6**: Differentially abundant (p < 0.05) archaeal/bacterial sequence variants colored and spread by taxonomic group in low compared to high hemlock woolly adelgid population levels across plant-associated habitats and host species. The x-axis is dimensionless and is used to spread apart microbial taxa. Sizes of points represent the average relative abundance of the SV in the low hemlock woolly adelgid population levels.

**Figure S7**: Relative read abundance of fungal classes across plant-associated habitats, hemlock woolly adelgid population levels, and host species.


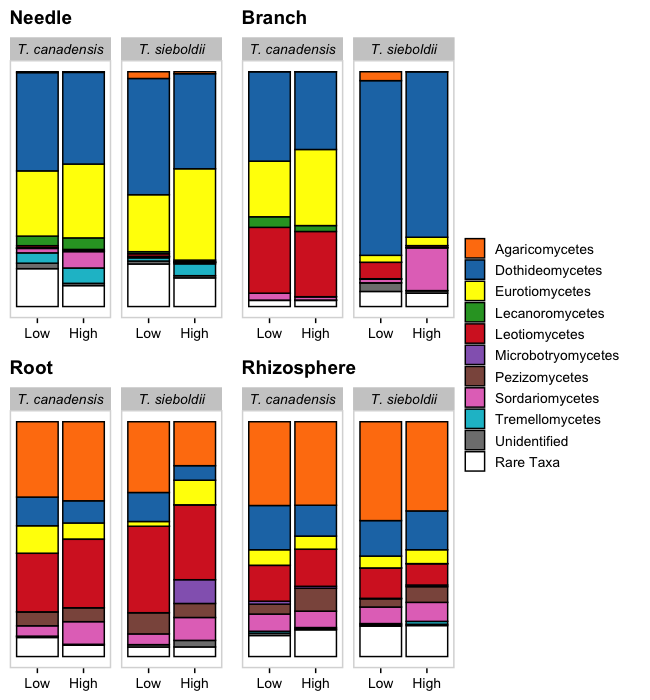


**Figure S8:** Relative read abundance of dominant fungal orders across plant-associated habitats, hemlock woolly adelgid population levels, and host species.

**
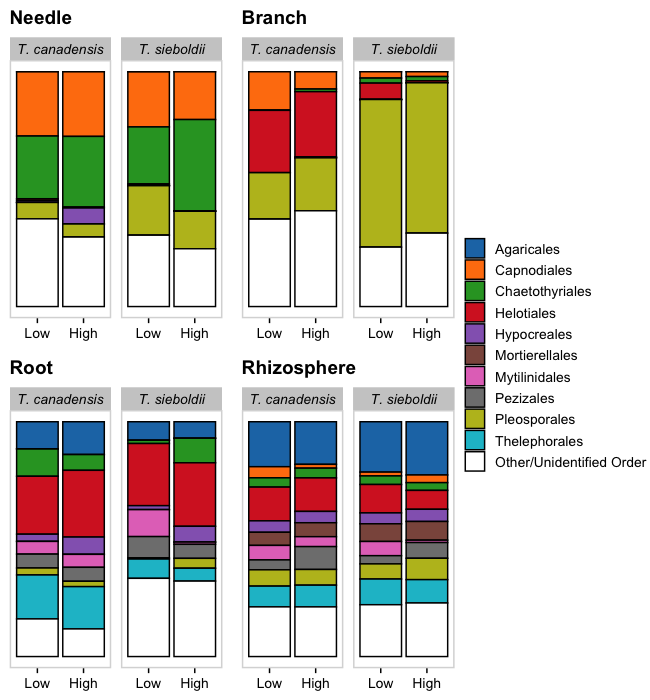
**

**Figure S9:** Relative read abundance of dominant fungal families across plant-associated habitats, hemlock woolly adelgid population levels, and host species.

**
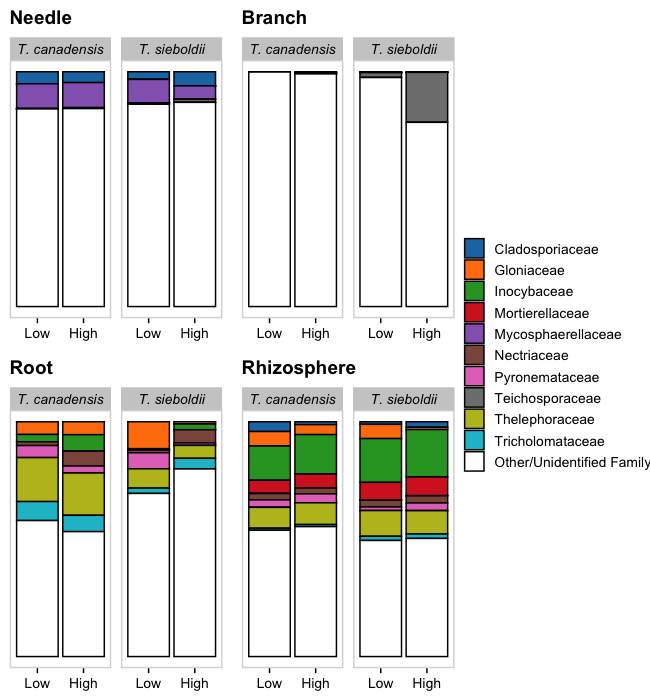
**

**Figure S10:** Relative read abundance of dominant fungal genera across plant-associated habitats, hemlock woolly adelgid population levels, and host species.

**
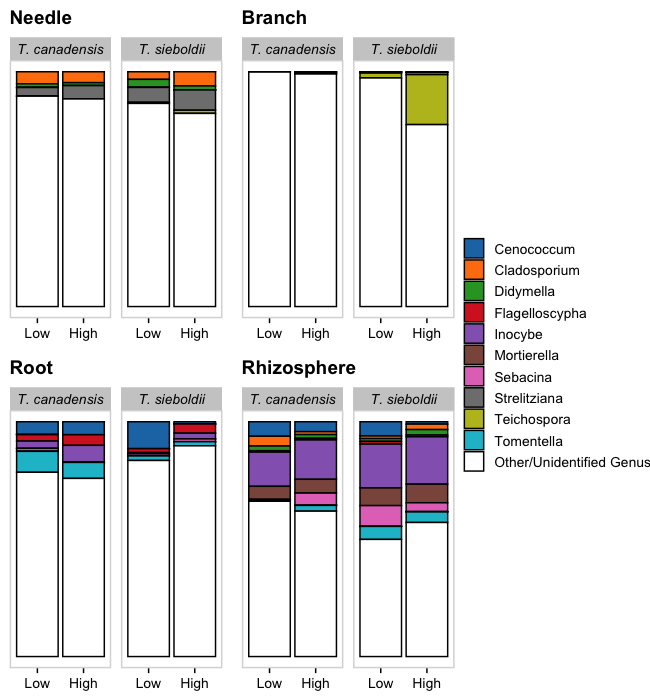
**

**Figure S11**: Differentially abundant (p < 0.05) fungal sequence variants (SV) colored and spread by phylum in low compared to high hemlock woolly adelgid population levels across plant-associated habitats and host species. The x-axis is dimensionless and is used to spread apart microbial taxa. Sizes of points represent the average relative abundance of the SV in the low hemlock woolly adelgid population levels.

**Figure S12**: Relative read abundance of ectomycorrhizal orders as a proportion of all ectomycorrhizal reads across belowground plant-associated habitats, host species, and hemlock woolly adelgid population levels.


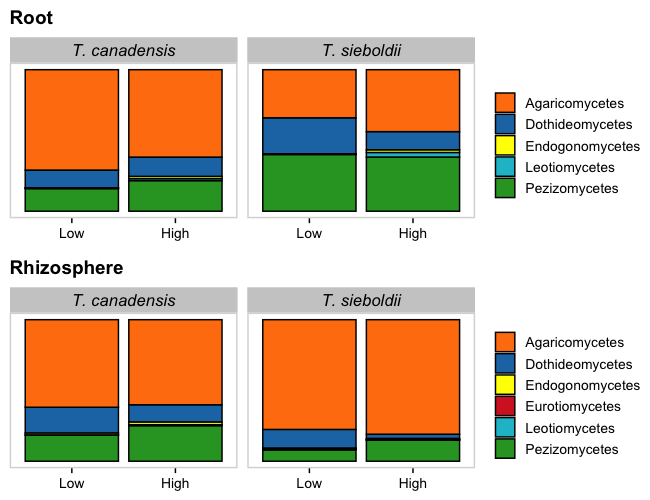

Supplement: Supplementary file 1 [file Data_Sheet_1.docx]
